# Sort, Assess, Life-Saving Intervention, Triage With Drone Assistance in Mass Casualty Simulation: Analysis of Educational Efficacy

**DOI:** 10.7759/cureus.10572

**Published:** 2020-09-21

**Authors:** Ethan N Hartman, Benjamin Daines, Christina Seto, Deborah Shimshoni, Madison E Feldman, Michelle LaBrunda

**Affiliations:** 1 Medicine, University of Central Florida College of Medicine, Orlando, USA; 2 Internal Medicine, University of Central Florida College of Medicine, Orlando, USA

**Keywords:** mass casualty, triage, drone, learner, global health, quality improvement

## Abstract

Introduction

Mass casualty incident (MCI) simulation and triage are educational methods used to provide high fidelity training to first response teams. Simulation and triage need to be as effective as possible to train professionals for true emergencies involving mass casualty. Although MCI simulation and triage have been used in the pre-professional setting (i.e. medical school, nursing school, etc.), more data is required regarding quality improvement of these simulations. This study focuses on quality improvement of MCI simulation and triage in the pre-professional training. In order to evaluate simulation quality to optimize future triage simulations, this study had three specific aims: (1) assess participant accuracy of triage after training in Sort, Assess, Life-Saving Interventions, Triage/Transport (SALT); (2) evaluate the role of stress and confidence in participants of triage simulation; (3) determine trainees’ perception of unmanned aerial vehicles (drones) in the setting of mass casualty simulation.

Methods

A total of 44 attendees of the University of Central Florida (UCF) College of Medicine Global Health Conference participated in this study across three groups. Each group was provided a 15-minute lecture on SALT protocol. After the training, the participants continued to a 30-minute simulation in which they were asked to accurately triage up to 46 patient-actors. Each participants’ triage designations were compared to the previously assigned designations of each patient-actor. Pre- and post-simulation surveys were collected and analyzed using Statistical Package for the Social Sciences (SPSS) (IBM Corp., Chicago, IL). All other data were analyzed using descriptive statistics.

Results

Qualitative and Likert data for the simulation were collected from 44 participants. Given a total of 1,113 triage scores (average of 25.29 triage designations per person), there was data to support that novice learners in this study tended to under-triage using the SALT protocol after 15-minute SALT training, with an overall accuracy of 52.43%. Survey data showed that confidence in mass casualty triage improved post-simulation, improving from median 3/10 to 5/10. Most participants were unaware of the use of unmanned aerial vehicles in MCI but most had positive opinions of their usefulness in MCI after the simulation, with a median score of 8/10.

Conclusions

Participant accuracy of triage after undergoing a 15-minute training in SALT triage was 52.43%, with a non-statistically significant tendency to under-triage. This accuracy level is consistent with other studies of SALT triage in MCI, but the tendency to undertriage requires further study for validation. Stress levels during the simulation were significantly elevated, while post-simulation confidence increased significantly from pre-simulation. The perception of drone utility in MCI was favorable among participants in this study, indicating drones may be useful for first response teams in future mass casualty simulations.

## Introduction

Mass casualty incident (MCI) simulation is an educational tool used by medical personnel (i.e., doctors, nurses, paramedics, etc.) to simulate true emergencies. Simulation allows medical personnel to practice emergency and first-response protocol in an appropriately stressful but safe environment [1-3]. These simulations often teach participants crucial skills such as triage and evoke important subjective feelings in participants such as stress. The quality of these MCI simulations, therefore, depends on both participants’ triage accuracy and subjective perceptions.

Mass casualty triage is a protocol for emergency response in scenarios of mass casualty when medical capacity may be overwhelmed [4]. MCI triage aims to reduce morbidity and mortality by stratifying patients based on injury severity, prioritizing patients with life-threatening injuries, and directing emergency resources such as supplies, hospitals, and medical personnel to patients appropriately [1,4]. One such triage protocol is the Sort, Assess, Life-saving intervention, Triage/Transport (SALT) protocol [5-7]. Other triage protocols include the Simple Triage and Rapid Treatment (START), Triage Sieve, and Careflight protocols [5-14]. SALT triage has been demonstrated to have higher accuracy than other types of triage protocols [15,16]. Hospitals and emergency response teams may use desired protocols based on their individual needs.

Studies of multiple triage protocols demonstrate variable levels of accuracy from one protocol to the next [8-16]. Variability inaccuracy is likely due to the types of errors made by first response teams [17,18]. One common error made by first response teams is “over-triage”, meaning to triage a victim’s injuries with higher severity than is medically indicated (i.e., over-utilization of resources) [19]. An example of over-triage is misinterpreting a patient’s simple abdominal contusion to be an aortic aneurysm, leading to unnecessary imaging and possibly unnecessary surgery. First response teams may also “under-triage” meaning they do not recognize the severity of a victim’s injuries. An example of under-triage is mistaking traumatic brain injuries like a concussion, which can lead to significant morbidity and mortality. Triage accuracy is an important measure of simulation quality because inaccuracies may lead to over-treatment or under-treatment, leading to wasted resources or increased mortality respectively [8,17-21].

Stress is an important aspect of simulation quality that correlates to overall educational experience and quality [22]. Mass casualty simulation is meant to be inherently stressful, which contributes to the overall realism of the training [2,5,22-24]. Although there are few data measuring stress during mass casualty simulation, it is probable that if an individual can triage accurately in a stressful simulation, the individual is more likely to triage accurately in a true emergency. Pedagogical studies have demonstrated that a small amount of anxiety is needed to perform well on performance exams, but too much anxiety was detrimental to performance. A similar principle has been noted in MCI simulation [22-24]. Few studies have examined self-reported levels of stress in mass casualty simulation. Understanding the extent of self-reported stress in participants may provide insight into the overall realism and therefore the quality of the simulation.

In the setting of MCI simulation triage, confidence among participants is a subjective measure of how accurately participants believe they can triage using a given triage protocol. Effective simulation increases confidence in participants’ ability to carry out simulation-specific tasks, and MCI simulation participants are more confident in their ability to respond to medical emergencies after MCI simulations [22,24]. A high-quality simulation is therefore one that gives participants the confidence to reproduce the skills they learn through simulation training.

Finally, unmanned aerial vehicles (hereby known as “drones”) are being used with increasing frequency in remote MCI and MCI simulation [25-28]. The advent of technology allows humanitarian organizations and researchers to deliver medical and food supplies to remote areas that are restricted by geography, natural disaster, or international conflict [29,30]. Because of their increasing use around the world, drones are becoming a popular method of surveillance and delivery, and thus are important to study in simulation.

MCI simulation is an evolving educational tool in the medical field, and medical professionals need to be well-trained to provide optimal aid to those in need. An effective simulation is therefore one that teaches triage accuracy, provides an appropriate amount of both stress and confidence for participants, and incorporates cutting edge technology, such as drones, to create a realistic and fruitful educational experience for the first response teams of tomorrow.

## Materials and methods

Setup

This prospective study was performed at the University of Central Florida (UCF) College of Medicine Global Health Conference on the main lawn on January 12, 2019. The lawn was monitored by the American Aviation Society, as the location was near a major international airport and included the flight path of several commercial airplanes. Permissions were obtained to fly the drones supplied by Archer First Response Systems for the duration of the simulation. This simulation was open to all participants of the conference, including medical students, nursing students, undergraduate students, and healthcare providers of all levels.

The simulation was conducted using 46 patient-actors and mannequins as victims. Each patient-actor was recruited from UCF College of Nursing and Lake Nona High School with personal and/or parental consent. In addition to patient-actors, this project utilized donated mannequins from the UCF College of Nursing and Nemours Children’s Hospital (Orlando, FL). The patient-actors and mannequins were designated according to the published SALT algorithm adapted from Lerner et al [5] into triage categories of minimal (13), delayed (14), intermediate (13), and expectant (6) depending on their injuries. Before the simulation, the researchers instructed patient-actors on severity-specific scripts. A trained team of moulage artists applied wound simulation make-up on each patient-actor according to their script and level of severity.

Simulations

**Figure 1:**
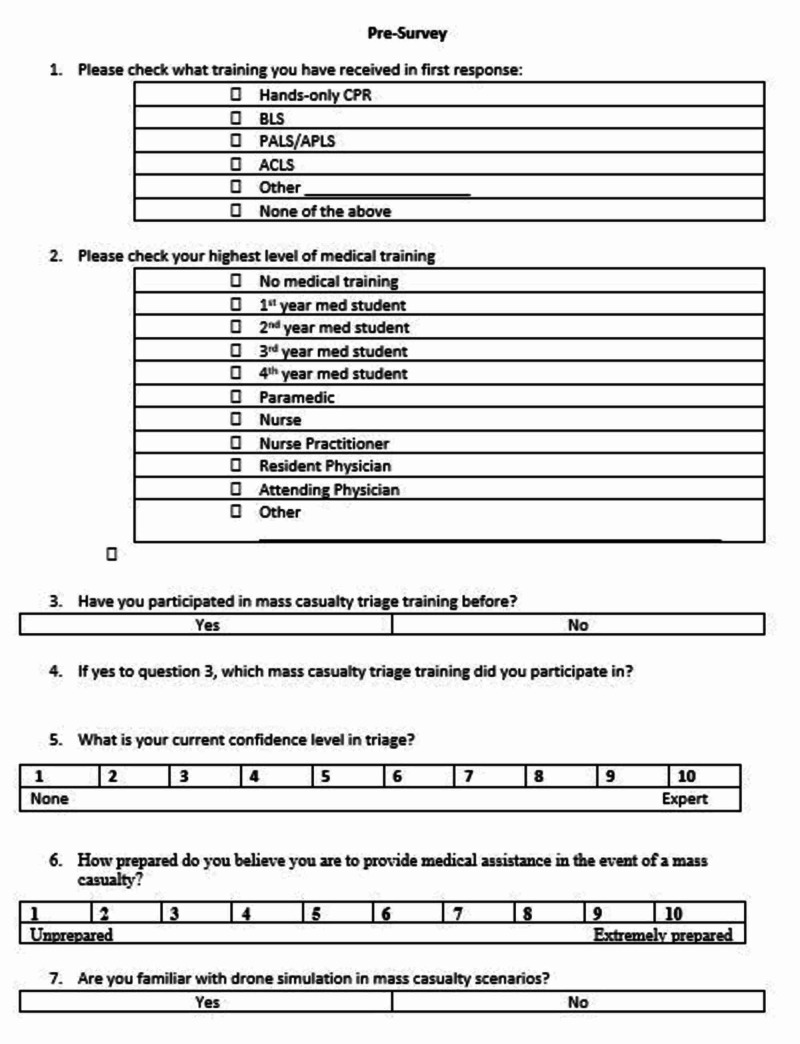
Pre-survey

**Figure 2:**
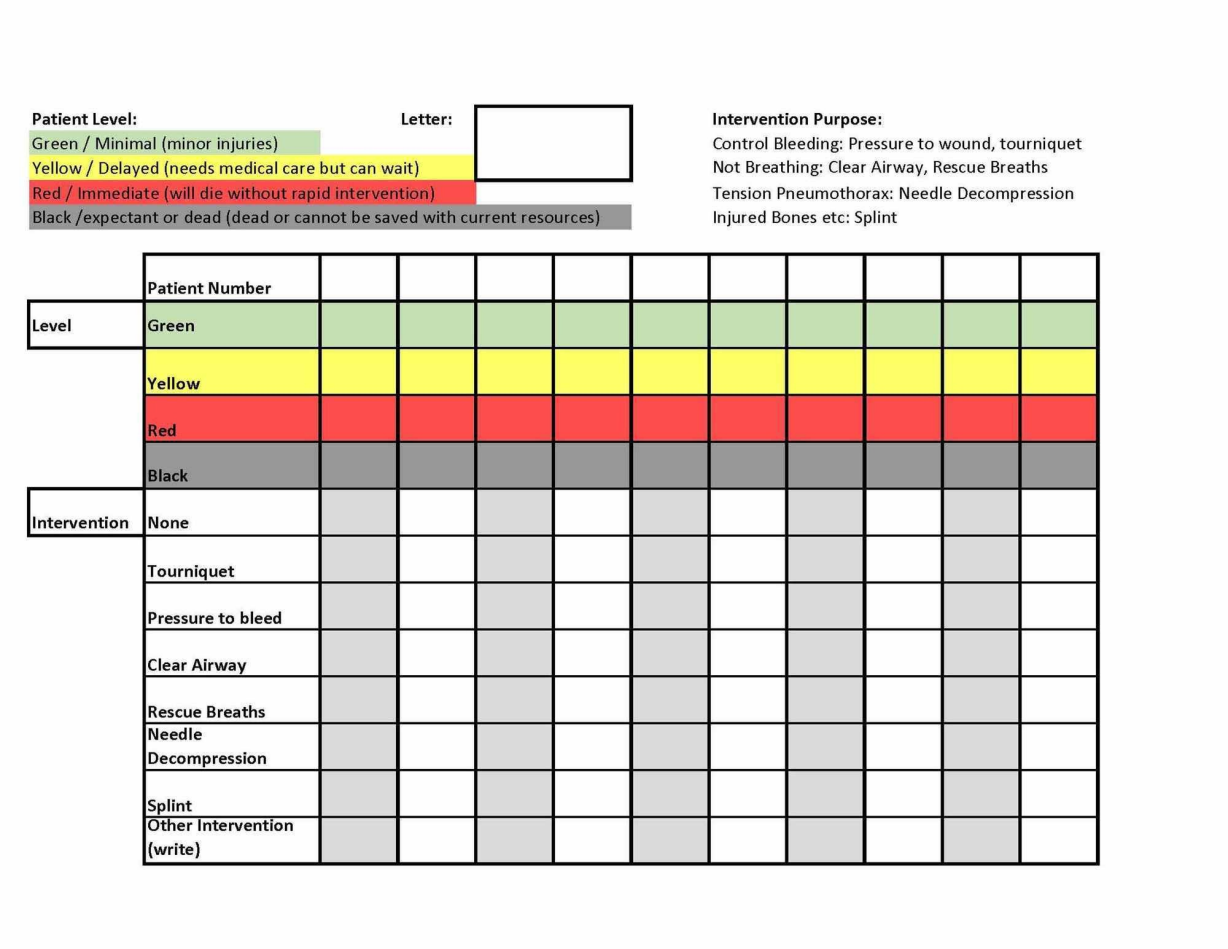
Triage form

**Figure 3:**
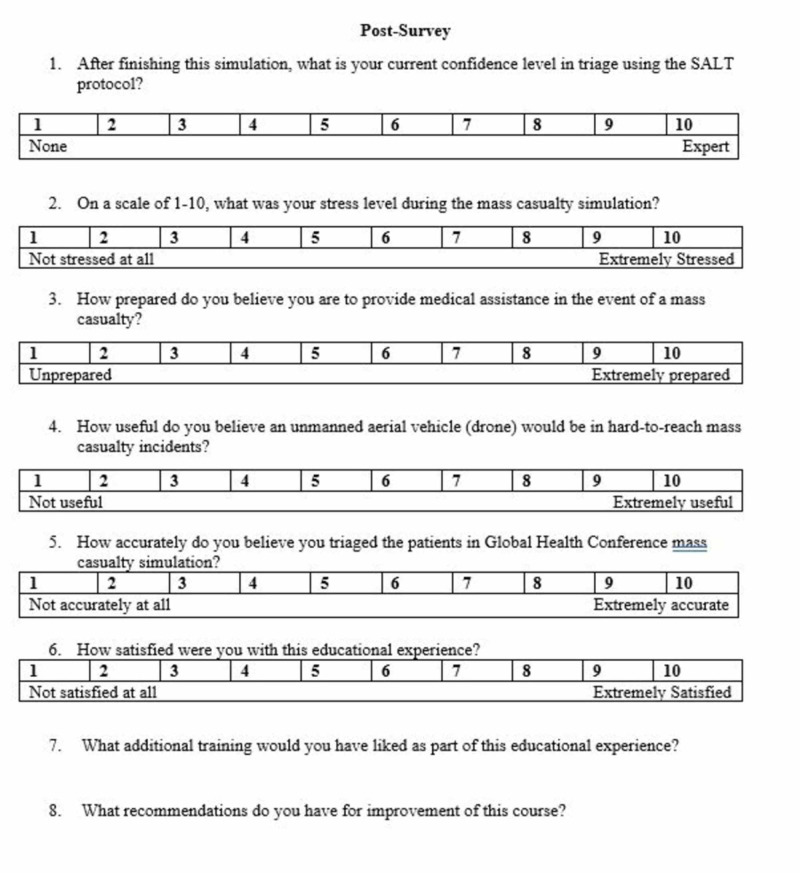
Post-survey

Participants were randomly assigned to one of three groups. Each study participant received a packet including an explanation of the research, a pre-simulation survey, a triage designation form, and a post-simulation survey (Figures 1-3). The explanation of research delineated the scope of the simulation as well as the data being collected. Participants completed a pre-simulation survey before the SALT training and simulation. Each group then received SALT training via a 15-minute PowerPoint lecture on SALT triage.

After the lecture, all participants engaged in a 30-minute simulation, during which they triaged as many patient-actors as possible during the allotted time using the triage designation form. During the simulation, earthquake noises played through a loudspeaker. Participants freely triaged and were not given specific instructions for the first 20 minutes of simulation. When 20 minutes had elapsed, an unmanned aerial vehicle arrived at the site of mass casualty, delivering tourniquets, automated external defibrillators, and Quick Clot. Participants provided care to the patient-actors using the delivered supplies for the final ten minutes of the simulation.

After the simulation, participants completed the post-simulation surveys. Researchers collected the post-simulation surveys and triage data and reset the scene for the next group. All data were collected de-identified, preserving the privacy and confidentiality of all participants.

Data acquisition

This study has two sources of data: (1) triage accuracy among participants and (2) survey responses. Descriptive statistics were used to measure triage accuracy among participants.

The survey was designed to assess participant stress, confidence, and perception of drones. The pre-simulation survey contained questions regarding previous training, confidence in triage/MCI, and awareness of drone use in MCI. The post-simulation survey included questions regarding confidence, stress, accuracy, and utility of drones in mass casualty simulation. Each question was rated on a 1-10 Likert scale, with 1 representing “strongly disagree” and 10 representing “strongly agree.” The post-simulation survey also had open-ended questions so participants could provide feedback in a free-response manner. Pre-simulation and post-simulation confidence levels were compared using a paired t-test, and other survey data were measured using descriptive statistics. All quantitative data used SPSS (IBM, Chicago, IL).

## Results

Forty-four participants completed triage designation forms. The percent accuracy, over-triage, and under-triage are demonstrated in Table 1.

**Table 1:** Average triage designations per participant. Percent correct, under-triage, and over-triage. Mean deviation from an accurate score.

Triage Category	Data
Average triage designations per participant	25.29
Percent correct	52.43%
Percent under-triage	31.36%
Percent over-triage	15.95%
Mean deviation from accurate score^*^	-0.199 (n=44)

The average number of triage designations per participant was 25.29, with a total number of triage designations of 1,113. The overall accuracy using the SALT triage algorithm was 52.43%, with a tendency to under-triage. Thirty-nine participants tended to under-triage, contributing to the negative average deviation from accurate triage score (0=accurate triage). Five participants tended to over-triage.

Forty-four participants completed pre-simulation surveys and 41 participants completed post-simulation surveys. Participants rated their pre-simulation confidence in using SALT triage as 3/10 on average, while post-simulation confidence was 5/10 on average. The difference between pre-simulation and post-simulation confidence in using SALT triage was statistically significant (p<0.05). In a similar measure of confidence, participants also rated their pre-simulation preparedness to assist in an MCI as 3/10 on average, while post-simulation preparedness to assist in an MCI, as well as replicate their triage skills, were both rated 5/10 on average (p<0.05). On a scale of 1 to 10, in which 1 represented “not useful” and 10 represented “extremely useful”, participants rated their simulation stress level to be a median 7/10 (mean 6.81). Participants rated their perception of drone utility as a median 9/10 (mean 8.67). Overall satisfaction of the educational experience is a median of 8/10 (mean 8.26), where 1 is not satisfied and 10 is extremely satisfied.

Participants were also given the opportunity for free-response comments on their post-simulation surveys. Comments were mainly focused on simulation improvement, and included suggestions for additional training, improved simulation design, and the inclusion of a post-simulation debrief. Out of the 44 participants, 18 requested additional training, such as more information regarding life-saving interventions (11), and more time for training before simulation (3). Participants also requested improvements in simulation design (6). 

## Discussion

This simulation provided an educational MCI workshop in a realistic but safe environment. This study evaluated quality based on the triage accuracy of the participants, perceived stress, perceived confidence levels, and perceived utility of drone use in MCI simulation. Also, comments solicited from the participants allowed for insight into participant experience.

An effective simulation is one in which participants learn to triage patients accurately. Although there is no accepted threshold for minimum accuracy, triage accuracy of approximately 52.43% is comparable to some studies of SALT triage and significantly less than other studies [10,13,15,16]. SALT remains one of the most consistent and accurate triage protocols used today, warranting further research to improve its quality and efficacy in simulation. 

A moderately high-stress level may provide a more realistic experience for those in simulation, as it allows students a safe but realistic scenario in which to train. The median perceived stress level in this study was moderately high (7/10), which is remarkable given the element of psychological and physical safety inherent to simulation. Elevated stress levels suggest that elements such as moulage, prosthetics, sound effects, time-bound practice, and patient-actors added a significant amount of urgency and realism that made the simulation stressful to participants. There is, therefore, evidence that this simulation was effective in its ability to create an environment in which students could practice their skills under duress.

In this study, confidence is defined as the perception of one’s own ability to triage appropriately using SALT protocol. As in all training, post-training confidence is an important marker of perceived training effectiveness [22-24]. Confidence improved significantly in participants from pre-simulation to post-simulation. Increased confidence following the simulation suggests that the brief didactic session combined with simulation was effective in training participants to be prepared for future MCI simulations.

Drones in MCI are useful for providing aid to remote areas, and this study’s utilization of drones illustrated their ability to deliver supplies to hard-to-reach or dangerous geographical areas. Participants perceived the drones to be exceedingly useful (9/10), likely because the drones delivered useful materials to the MCI vicinity, allowing participants to have access to life-saving interventions necessary for adequate treatment of patient-actors (e.g., tourniquets, automated external defibrillators, QuikClot). Several factors contribute to the utility of drones in MCI simulation: (1) proximity of supplies, (2) short time elapsed to load the drone with supplies, (3) short time elapsed to deploy the drone, (4) convenient location to which the supplies are deployed, and (5) the necessity of supplies deployed by the drone. Drones in MCI simulation were exceedingly useful to participants and should be incorporated into future MCI simulation as they are becoming increasingly popular and prevalent.

Limitations

While simulations are designed to approximate reality, they are not perfect representations of real-life scenarios. Constraints on both resources and time in this simulation may have limited the learning experience for participants. Although most participants noted no first response or medical training, a baseline test was not done to determine participants’ proficiency in triage before the simulation. It is difficult to control for inherent differences in patient-acting (i.e. over-acting or under-acting) that may have contributed to variations in triage. Lastly, small sample size and self-selection bias may have led to errors in assessment due to systemic differences between participants and nonparticipants.

Future directions

MCI simulations will continue to be a part of the UCF College of Medicine Global Health Conference, incorporating moulage, SALT triage, and suggested improvements to the simulation. In addition, triage accuracy scores can be assessed each year to assess trends and consistency of SALT triage. Pre-simulation and post-simulation surveys will continue to provide insight into participant perception. Continuing cohesive relationships with Archer First Response Systems and Simetri moulage companies will enhance the incorporation of drones and high-fidelity make-up for patient-actors adding to the realism of the simulation. 

## Conclusions

The accuracy of SALT triage post-training was found to be 52.43% with a tendency to under-triage, which is consistent with other studies of SALT. Improvements to the simulation design, including more time for formal triage training as well as debrief, may lead to improvements in SALT triage skills and thus this accuracy level. High-stress level reported by participants demonstrates simulation efficacy in that the simulation was a realistic environment for practicing MCI triage skills. Confidence improved from pre-simulation to post-simulation, demonstrating that the pre-simulation and intra-simulation training was effective in this MCI simulation. The drone technology was perceived as useful for participants due to the delivery of supplies for life-saving interventions as defined by SALT guidelines. 
